# Early ascending growth is associated with maternal lipoprotein profile during mid and late pregnancy and in cord blood

**DOI:** 10.1038/s41366-023-01361-x

**Published:** 2023-08-17

**Authors:** Elina Blanco Sequeiros, Anna-Kaisa Tuomaala, Rubina Tabassum, Paula H. Bergman, Saila B. Koivusalo, Emilia Huvinen

**Affiliations:** 1 University of Helsinki and Helsinki University Hospital, Helsinki, Finland; 2 Soite Children’s Hospital, Kokkola, Finland; 3 Department of Pediatrics, University of Helsinki and Helsinki University Hospital, Helsinki, Finland; 4 Institute for Molecular Medicine Finland, HiLIFE, University of Helsinki, Helsinki, Finland; 5 Biostatistics Consulting, Department of Public Health, University of Helsinki and Helsinki University Hospital, Helsinki, Finland; 6 Department of Obstetrics and Gynecology, University of Turku and Turku University Hospital, Turku, Finland; 7 Department of Obstetrics and Gynecology, University of Helsinki and Helsinki University Hospital, Helsinki, Finland

**Keywords:** Obesity, Biostatistics

## Abstract

**Introduction:**

Intrauterine conditions and accelerating early growth are associated with childhood obesity. It is unknown, whether fetal programming affects the early growth and could alterations in the maternal-fetal metabolome be the mediating mechanism. Therefore, we aimed to assess the associations between maternal and cord blood metabolite profile and offspring early growth.

**Methods:**

The RADIEL study recruited 724 women at high risk for gestational diabetes mellitus (GDM) BMI ≥ 30 kg/m^2^ and/or prior GDM) before or in early pregnancy. Blood samples were collected once in each trimester, and from cord. Metabolomics were analyzed by targeted nuclear magnetic resonance (NMR) technique. Following up on offsprings’ first 2 years growth, we discovered 3 distinct growth profiles (ascending *n* = 80, intermediate *n* = 346, and descending *n* = 146) by using latent class mixed models (lcmm).

**Results:**

From the cohort of mother-child dyads with available growth profile data (*n* = 572), we have metabolomic data from 232 mothers from 1st trimester, 271 from 2nd trimester, 277 from 3rd trimester and 345 from cord blood. We have data on 220 metabolites in each trimester and 70 from cord blood. In each trimester of pregnancy, the mothers of the ascending group showed higher levels of VLDL and LDL particles, and lower levels of HDL particles (*p* < 0.05). When adjusted for gestational age, birth weight, sex, delivery mode, and maternal smoking, there was an association with ascending profile and 2nd trimester total cholesterol in HDL2, 3rd trimester total cholesterol in HDL2 and in HDL, VLDL size and ratio of triglycerides to phosphoglycerides (TG/PG ratio) in cord blood (*p* ≤ 0.002).

**Conclusion:**

Ascending early growth was associated with lower maternal total cholesterol in HDL in 2nd and 3rd trimester, and higher VLDL size and more adverse TG/PG ratio in cord blood.

**Clinical trial registration:**

ClinicalTrials.gov, http://www.clinicaltrials.com, NCT01698385.

## Introduction

Childhood obesity is a major public health challenge [1] affecting countries all around the world. It is estimated that about half of school-age children who have obesity will continue to have obesity as adults [2]. Obesity can profoundly affect both physical and mental health of these children - it is associated with poor social and emotional well-being, lower self-esteem, poor academic performance, and a lower quality of life [3–5]. Despite extensive research, there is neither easy nor efficient treatment at hand [6]. The focus should therefore be on preventive measures, and thus increasing our knowledge of early mechanisms preceding childhood obesity could act as a cornerstone for developing new strategies.

Growth is a dynamic process depicting individual potential and health. Recent studies on early programming of childhood obesity [7] have shown that maternal factors, e.g., high maternal BMI and gestational diabetes (GDM) are strongly associated with excess fetal growth, adiposity, and increased risk of cardiometabolic morbidity and insulin resistance [8–11]. In addition to fetal growth and birth weight, also early growth trajectories seem to be relevant [12, 13]. In the RADIEL study, ascending early growth was associated with childhood adiposity, independent of the child’s own lifestyle and confounding maternal factors [14]. Highlighting the role of intrauterine conditions, also early growth has been associated with maternal obesity and other pregnancy-related factors. [15, 16]. The mechanisms underlying these associations, however, are not well understood.

Although evidence linking intrauterine conditions to later offspring health is already abundant, quite little is known about the influence of maternal metabolomics on future health of the offspring [17]. A few studies focusing on associations between the 2nd trimester or cord blood metabolomics and early childhood overweight/obesity risk have found associations with xenobiotics, lipids, methyl donors, and metabolites related to tryptophan [18, 19]. Interestingly, Zhao et al. [18] found associations between maternal metabolomics in 2nd trimester and adverse early growth trajectories, while Cao et al. discovered a link between cord blood metabolomics and persistent obesity into adolescence [20]. To our knowledge, there are, however, no studies assessing maternal longitudinal metabolite profiling during pregnancy and their possible underpinning role in early growth trajectories leading to childhood obesity.

Therefore, the aim of this study was to assess whether the metabolite profile either during pregnancy or in cord blood could be one possible link between intrauterine conditions and early growth. We investigated whether maternal metabolomics in three time points during pregnancy and fetal metabolomics in cord blood associate with longitudinal early growth trajectories of offspring. Our special focus was on the ascending early growth, which has been previously associated with childhood adiposity.

## Methods

### Study design

This is a secondary analysis of the RADIEL study, a multi-center, randomized controlled intervention trial aiming at prevention of GDM through lifestyle modification. The study enrolled a total of 724 women at high diabetes risk, in the Helsinki Metropolitan area or in Lappeenranta, Finland, during years 2008 to 2011. The recruited women had a history of GDM and/or obesity (BMI ≥ 30 kg/m^2^). Exclusion criteria were age < 18 years, current type 1 or 2 diabetes, medication-altering glucose metabolism, multiple pregnancy, severe psychiatric problems, physical disabilities, substantial communication difficulties, and current substance abuse. The participants were randomized to a control group or to a combined lifestyle intervention group, emphasizing dietary goals following Finnish nutrition guidelines, physical activity (150 min/week), and limiting gestational weight gain. During pregnancy, the study visits took place once in each trimester and 6 weeks, 6 and 12 months after delivery. Previous publications have provided detailed information on the study design and methods [21–23].

The study complies with the Declaration of Helsinki and was approved by the Ethical Boards of Helsinki University Hospital (HUS) and South-Karelia Central Hospital (SKCH). All the participants entered the study voluntarily and gave written informed consent. They were also free to discontinue at any point.

### Measurements

#### Pregnancy

Every study visit included anthropometric measurements (height, weight, and blood pressure) and blood samples for analyzing markers of glucose and lipid metabolism as well as inflammatory markers. A 2-hour 75 g oral glucose tolerance test (OGTT) was performed at enrollment (if before pregnancy), in the first and second trimesters of pregnancy, and 6 weeks and 12 months postpartum. GDM diagnosis was based on one or more pathological values in the OGTT (normative values: 0 h < 5.3 mmol/l, 1 h < 10.0 mol/l, and 2 h < 8.6 mmol/l). Prepregnancy weight was self-reported for those recruited in early pregnancy and the weight at the last visit before pregnancy for those recruited before pregnancy. Gestational weight gain (GWG) was defined as the difference between prepregnancy weight and weight at the third-trimester study visit.

Questionnaires provided data on maternal years of education and lifestyle such as substance use (smoking, alcohol) and moderate-intensity physical activity (PA) (min/week). Food frequency questionnaires (FFQs) offered information for calculating a Healthy Food Intake Index (HFII), which served as a description of maternal diet as a whole [24]. Maximum score was 18 and fulfilling nutritional goals contributed points as follows: fast food (0–1 points), bread fat spread (0–2 points), low-fat cheese (0–1 points), intake of high-energy/low-nutrient snacks (0–2 points), sugar-sweetened beverages (0–1 points), high-fiber grains (0–2 points), low-fat milk (0–2 points), fish (0–2 points), red and processed meat (0–2 points), vegetables (0–2 points), and fruits and berries (0–1 points). A higher score indicated a healthier diet.

#### Offspring

The data from birth such as date of delivery, gestational weeks at delivery, delivery mode and placental weight were obtained from the hospital birth records. They also provided data on newborn characteristics at birth (weight, length, head circumference, and Apgar points). Cord blood samples from umbilical vein were collected at delivery for metabolomics analyses.

Communal childcare clinic registries from first 2 years of life provided additional data including growth (weight, height) and information on early feeding: total duration of breast feeding and exclusive breastfeeding, and the age at introduction of solid foods.

Our previous study used latent class mixed modelling and identified three distinct growth profiles (ascending *n* = 80, intermediate *n* = 346, and descending *n* = 146) based on the development of ponderal index (PI) during the first two years of life [12].

### Metabolite Profiling

Metabolite analysis was performed using high-throughput proton NMR metabolomics platform process (Nightingale Health Plc, Helsinki, Finland) from fasting plasma samples, which were separated immediately and stored at −80 °C. Only the metabolites detected in >70% of the participants were included in the analyses. Thus, 220 metabolites in each trimester and 70 metabolites from cord blood were included in this study. These metabolites cover multiple metabolic pathways, including lipoprotein lipids and subclasses, apolipoproteins, fatty and amino acids, ketone bodies, glycolysis and gluconeogenesis-related metabolites, fluid balance, and inflammation. Details of the experimentation and applications of the NMR metabolomics platform have been described previously [25, 26]. In brief, 260 μL of serum were carefully mixed with sodium phosphate buffer (260 μL) and moved to NMR tubes. PerkinElmer JANUS Automated Workstation equipped with an 8-tip dispense arm with Varispan was used to handle all the liquid serum samples. Nightingale Health laboratory setup is a combination of Bruker AVANCE III 500 MHz (a selective inverse room temperature probe head) and Bruker AVANCE III HD 600 MHz spectrometers (a cryogenically cooled triple resonance probe head; CryoProbe Prodigy TCI), both with the SampleJet robotic sample changer. Lipoprotein and low-molecular-weight metabolites data was automatically collected either with the 500 MHz or the 600 MHz spectrometer using a standard parameter set. The lipoprotein extraction procedure was done manually with Integra Biosciences VIAFLO 96 channel electronic pipette. Then lipid extracts were moved into the NMR tubes and data was collected using 600 MHz instrument with standard parameter set. NMR spectra was Fourier transformed and underwent numerous automated spectral processing steps and quality checks. The NMR platform has been used in large-scale epidemiological samples and in samples of pregnant and non-pregnant women [27]. The cord blood metabolomics platform became available in 2019.

### Statistical analysis

The data are presented as means with standard deviations (SD), medians with interquartile range (IQR), or as frequencies with percentages. The Shapiro–Wilk test was used to examine normal distribution of the variables. The Fisher’s test, Mann–Whitney *U* test, Kruskal-Wallis test, Chi-square test, ANOVA, or the independent samples T-test were used for between-group comparisons, as appropriate. We excluded from the analysis extreme outliers (deviating 1.5 times IQR from Q1 and Q3). Metabolites were log-transformed to achieve normal distribution. Principal component analysis (PCA) was used to assess the overall variability in the metabolomic data and 25 PCs explained 95% of the variance in the dataset. After correcting for multiple testing, associations with *p* < 0.002 (0.05/25 to account for 25 PCs) were considered significant.

Logistic regression analysis was used to assess associations between metabolites levels (maternal and cord blood) and growth trajectories. The analyses were adjusted for gestational age, birth weight SD (standardized for gestational age), gender, delivery mode, and maternal smoking. Mixed model was used for studying the longitudinal associations between metabolomics throughout pregnancy and early growth, and these analyses were also adjusted for gestational age, birth weight SD, gender, delivery mode, and maternal smoking. All analyses were performed with the SPSS 24.0 software program (SPSS Inc., Chicago, IL, USA) and figures produced with R studio 2022.07.1.

## Results

**Fig. 1:**
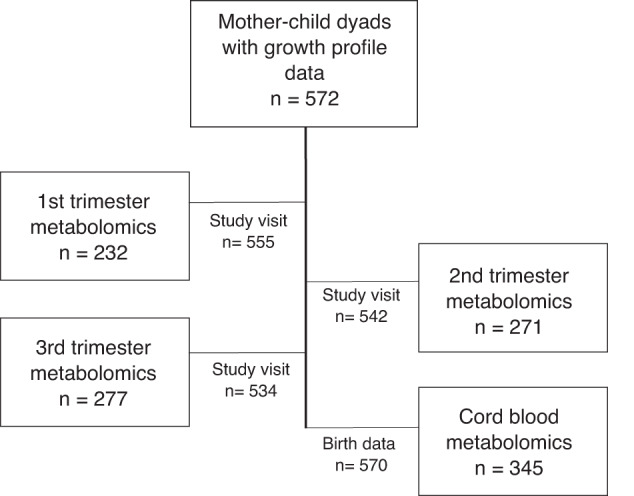
Flow chart of the study. Number of metabolomics data and study visits in each trimester. Number of birth data and cord blood metabolomics data.

From the cohort of mother-child dyads with available growth profile data (*n* = 572), we have metabolomics data from 232 mothers from the 1st trimester, 271 from the 2nd trimester, 277 from the 3rd trimester and 345 from cord blood (Fig. 1). Mean BMI of the mothers was 31.5 kg/m^2^ and altogether 270 out of 558 (47%) had GDM. Table 1 presents the characteristics of the participants and their children according to the growth trajectory. There were statistically significant differences between the groups in maternal pre-pregnancy BMI, smoking during pregnancy, early GDM, gestational age at delivery, and offspring size at birth (Table 1). There were no differences between the groups in maternal lifestyle during pregnancy or in gestational weight gain.

**Table 1:** Characteristics of the participants and their children according to the growth trajectory.

Variable	Ascending group	Intermediate group	Descending group	*P* value
Mother				
Age in 1st trimester, years	31 (4.6)	32 (4.6)	32 (4.3)	0.289
Prepregnancy BMI, kg/m^2^	32.8 (5.8)	31.3 (5.9)	30.5 (5.5)	0.030
GWG, kg	9.2 (5.9)	8.3 (5.7)	8.7 (6.0)	0.561
Years of education, years	15 (12, 15)	15 (12, 17)	15 (12, 17)	0.253
PA in 1st trimester, min/week	60 (30, 100)	60 (30, 140)	60 (30, 160)	0.496
PA in 2nd trimester, min/week	60 (30, 140)	60 (30, 120)	70 (45, 120)	0.618
PA in 3rd trimester, min/week	30 (0, 90)	60 (0, 120)	38 (0, 104)	0.466
HFII in 1st trimester, points	10 (7, 12)	10 (8, 12)	10 (9, 12)	0.480
HFII in 2nd trimester, points	10 (8, 12)	11 (8, 12)	11 (9, 13)	0.175
HFII in 3rd trimester, points	10(8, 12)	11 (8, 13)	11 (9, 12)	0.103
GDM, *n*	23 (34.8)	121 (46.2)	56 (47.5)	0.204
Cesarean section, *n*	22 (33.3)	54 (20.3)	23 (19.5)	0.054
Smoking in pregnancy, *n*	7 (12.5)	11 (4.6)	3 (2.8)	0.025
Child				
Gestational age at birth, weeks	39.2 (2.5)	40.0 (1.3)	40.1 (1.4)	0.001
Birth weight, g	3407 (661)	3682 (472)	3840 (488)	< 0.001
Birth weight SD	−0.09 (1.08)	0.23 (0.94)	0.55 (0.94)	< 0.001
Birth length SD	0.07 (1.16)	0.10 (0.99)	0.20 (0.95)	0.620
Sex (girls), *n*	36 (54.5)	124 (46.6)	55 (46.6)	0.492

**Fig. 2:**
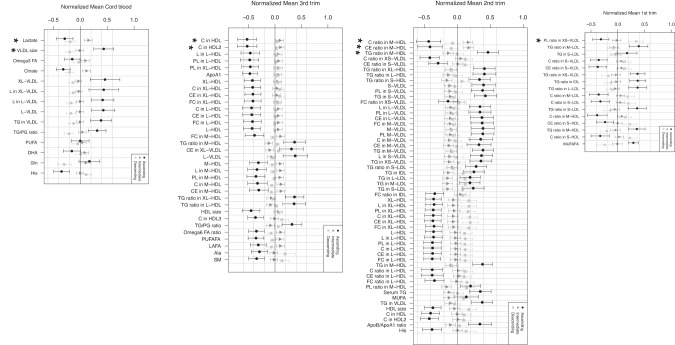
Normalized mean levels of the metabolites in each trimester and in cord blood. The plots show only the metabolites with *p* < 0.05 and those with *p* ≤ 0.002 are marked with *.

When comparing the mean levels of the metabolites in three different growth trajectories (ascending *n* = 80, intermediate *n* = 346, and descending *n* = 146), we discovered that in each trimester of pregnancy, there were differences between the groups in very low-density lipoprotein (VLDL) and low-density lipoprotein (LDL) particles, as well as in high-density lipoprotein (HDL) particles (*p* < 0.05) (Fig. 2). In cord blood, on the other hand, there were differences in the mean VLDL size and levels of lactate. The supplementary tables [1–4] provide the mean levels of the metabolites with most significant differences (*p* < 0.05) between the groups.

**Fig. 3:**
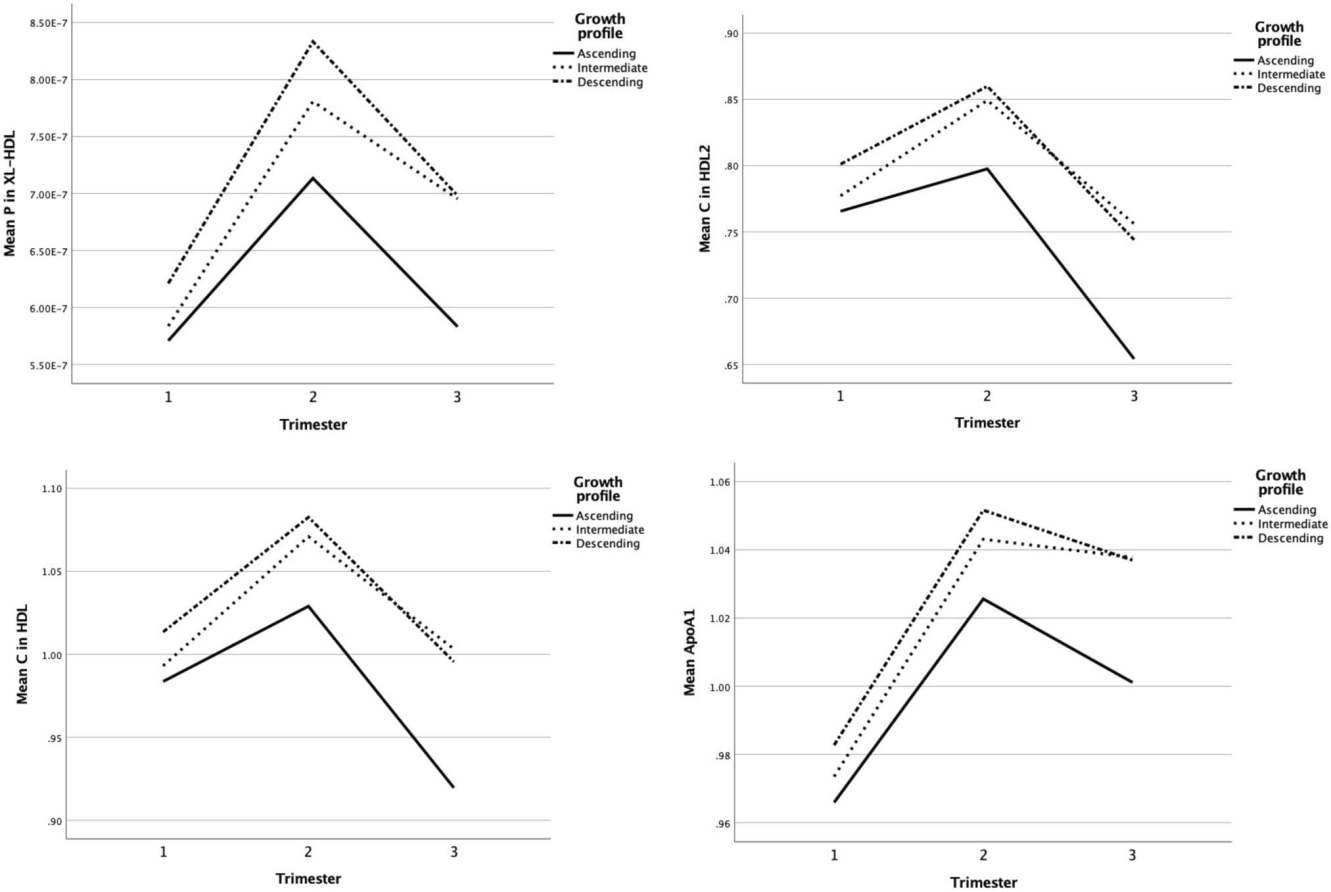
Longitudinal associations between the most significant metabolites with growth profiles. Concentration of XL-HDL (*p* = 0.002), cholesterol in HDL2 (*p* = 0.002), cholesterol in HDL (*p* = 0.003), and Apolipoprotein A1 (*p* = 0.005), adjusted for gestational age, birth weight SD, gender, delivery mode, and maternal smoking.

Longitudinal assessment of distinct metabolites throughout the pregnancy also yielded significant differences in HDL2 and in very large HDL between the growth profiles (Fig. 3). We also assessed the AUC of each metabolite, but there were no significant associations with growth profiles and neither did the change in the metabolites between specific trimesters during pregnancy reach any statistical significance.

**Fig. 4:**
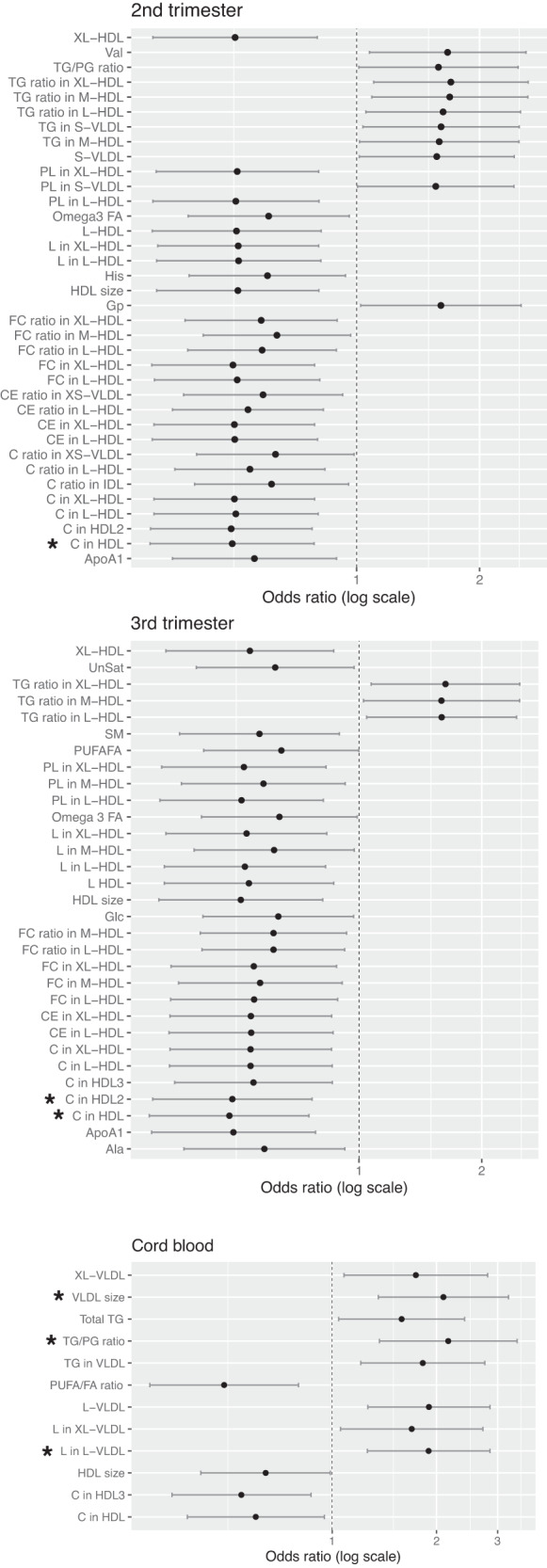
Associations between the metabolites in the 2nd and the 3rd trimesters and cord blood with the ascending growth profile, adjusted for gestational age, birth weight SD, gender, delivery mode, and maternal smoking. Forest plots show the metabolites with *p* < 0.05, descending profile as the reference, and * marks the ones with *p* ≤ 0.002 for statistical significance after correction for multiple testing.

In the logistic regression analysis, ascending growth profile was associated with several metabolites (Fig. 4). None of the metabolites in the 1st trimester showed statistically significant associations with growth profiles, but in the later half of the pregnancy, e.g. lower levels of several HDL particles and ApoA1were associated with adverse growth (*p* ≤ 0.005). In the cord blood, on the other hand, higher levels of VLDL particles and triglycerides were associated with the ascending growth profile. Specifically, after correction for multiple testing, there were associations with the 2nd trimester lower total cholesterol in HDL2, 3rd trimester lower total cholesterol in HDL2 and in HDL, as well as larger VLDL size and higher ratio of triglycerides to phosphoglycerides (TG/PG ratio) in cord blood (*p* < 0.002) (Fig. 4).

## Discussion

This study demonstrates an association between lipoprotein profiles during pregnancy and in cord blood with early growth of the offspring. We show that ascending growth, which was associated with later childhood adiposity in our previous study, was associated with lipoprotein composition – lower cholesterol in HDL and HDL2 in mid and late pregnancy, as well as higher VLDL size and triglycerides to phosphoglycerides ratio in cord blood. Our results suggest that the association between offspring’s growth and maternal lipoprotein profile during pregnancy is dependent on the stage of pregnancy.

Metabolite profile changes during pregnancy [28]. In a normal pregnancy, there is an increase for example in lipid, lipoprotein, and apolipoprotein levels and it is estimated that total cholesterol levels increase by 43%, LDL by 36%, HDL by 25%, and TG by 2.7-fold [29]. NMR-based metabolomics analyses have revealed changes in certain amino acids and fatty acids compared to non-pregnant women [30]. In pregnancies complicated with maternal obesity or GDM, there are more adverse metabolite measures and women with obesity seem to start their pregnancy already in a more deranged state, having less room for change throughout pregnancy [28, 31–33]. Recent reports from Zhao et al. and Cao et al. concerning maternal 2nd trimester and cord blood metabolomics and offspring early growth suggested that the use of metabolite profiles might be clinically important in predicting later overweight or obesity of offspring [18, 20]. These prior results were promising, but unfortunately metabolomics were measured only at one time point. According to our results, especially the lipid profile in the 2nd and 3rd trimester was associated with offspring growth. Interestingly Gademan et al. also found that maternal lipid profile based on standard enzymatic and turbidimetric technique in early pregnancy has been connected to offspring adiposity at 5–6 years of age, independent of pre-pregnancy maternal BMI [34]. Overall, the association between offspring’s growth and maternal metabolomics during pregnancy seems to be dependent on the stage of pregnancy.

Previous studies have suggested that higher VLDL and lower HDL are tied closely to obesity [35] and our results are well in line, demonstrating an association between levels of several HDL and VLDL subclasses in the second and third trimesters of pregnancy with offspring’s’ growth trajectories. HDL mainly consists of glycerophospholipids, cholesteryl esters (ChoE), sphingomyelins, and triacylglycerols, but the role of different particle ratios in HDL are not yet fully understood [36]. HDL removes excess cholesterol from tissues and has for that reason been traditionally linked to lower risk of cardiovascular disease. Especially higher HDL subfraction type2 (HDL2) levels are associated with lower risk of myocardial infarction [37]. More recent studies have, however, shown, that there are also many other functions in HDL as inhibiting inflammation, reducing oxidative stress, maintaining immune and cardiovascular health [38]. Suleiman et al. pointed also that HDL has an important role in pregnancy adaptation [39]. Altered HDL metabolism and low HDL2 levels have been reported in hypertensive mothers and this has associated with being small-for-gestational age (SGA) in newborns, probably due to excessive inflammation and oxidative stress [38, 40]. SGA could lead to adverse health outcomes after birth if rapid catch-up growth follows [41].

We also discovered a difference in the levels of ApoA1 between the growth trajectories. ApoA1 is an integral part and the major structural protein of HDL, mediating the many of antiatherogenic functions of HDL [38]. When assessing the longitudinal measures of metabolites during pregnancy, our results demonstrated an association between HDL2 and very large HDL and the adverse growth profile. Our results can be considered in line with prior results as also in our study HDL and VLDL were associated with the adverse growth profile associated with childhood obesity, suggesting that metabolite profile during pregnancy is a continuum.

Fatty acids are essential for fetal growth [42]. In our study pregnancies leading to adverse offspring growth showed lower mean levels of several unsaturated fatty acids in the 3rd trimester. Fatty acids are strongly correlated with dietary intake and interestingly we have previously shown that maternal dietary n-3 PUFA intake during pregnancy was associated with offspring adiposity at 5 years of age [43]. This has been investigated also in a RCT, where omega-3 supplementation for women with obesity improved fetal growth in lean mass and longer gestation [44]. Outside pregnancy Omega-3 PUFA supplementation was associated with increase in large HDL2 and a decrease in small HDL3 [45]. In our study, maternal physical activity and diet did not differ between the distinct growth profile groups, but the gross measurement methods could naturally offer a possible explanation.

However, there are also other mechanisms in addition to fetal growth which could be mediating the association between maternal lipoprotein profile and childhood growth. These metabolites have a capacity to influence epigenetic mechanisms and also the maturation of adipocytes of the offspring. Interestingly, elevated ApoA1 has even been connected to offspring eating behavior such as slowness of eating and lower enjoyment of food, as well as lower energy, fat and carbohydrate intake at the age of 5 years [46]. This could provide an explanation for the lower levels of ApoA1 associating with ascending growth in this study and also match our earlier results concerning the association between ascending growth and later childhood adiposity.

The major strength and novelty of our study is combining longitudinal analysis of maternal metabolomics at all three trimesters during pregnancy as well as in cord blood to offspring’s growth measured at several time points during the first two years of life. Our study population consists of a large cohort of well-characterized pregnancies. We have measured metabolomics in three different stages during pregnancy as well as from cord blood and this enables us to focus on distinct time-points and their importance for offspring health, considering the constantly changing metabolomics during pregnancy. We also have maternal anthropometrics and lifestyle recorded at each trimester of pregnancy. From the offspring, we have accurate and repeated measurements performed by professional healthcare workers until the age of 2 years, which enabled us to do longitudinal growth trajectory analysis.

Our study is, however, not without limitations. The fact that the study population consisted of only high-risk pregnancies and all Caucasian origin, limits naturally the generalizability of our results. In this study, we also relied on rather general dietary data, a HFII calculated from a simple FFQ, and naturally taking advantage of e.g., 3-day food diaries would have given a more detailed view of the macronutrient intake. Nevertheless, we believe that this detailed analysis of lifestyle factors would be out of the scope of this article. We also performed a targeted metabolomics analysis and therefore have only a limited number of metabolites and even less from the cord blood. The panel used is also very lipid centric. When considering growth, however, lipids have been proven very crucial and therefore this specific panel seems justified. Unfortunately, the study protocol did not include measurements of newborn body composition which would have been an interesting addition to our analyses. We found a statistically significant difference between the groups in maternal smoking, which is also a known risk factor for childhood obesity [47] Smoking may also affect lipoprotein levels by increasing total cholesterol, VLDL, LDL and TG, as well as by lowering HDL and ApoA1 [48] and this might have impacted our results.

Maternal obesity and GDM are strongly associated with childhood obesity, but despite many studies the underlying mechanism remains still unclear [11, 49]. In our study, all the participants were high-risk women with either GDM history or obesity, but our focus was on the metabolic milieu during pregnancy, independent of the underlying factors such as GWG, GDM, and/or adiposity. Future studies are of course needed to further assess the association between modifiable antenatal factors and maternal metabolite profile during pregnancy.

The origin of childhood obesity is multifactorial, including both antenatal [50] and postnatal conditions [51] as well as genetics [52]. Our results show that intrauterine conditions are important, and we might have an opportunity for prevention of childhood obesity as early as in the fetal period. In the optimal situation, the maternal metabolomics during pregnancy could help us to identify children needing intensified follow-up and possible support for the whole family. Future research should focus on the possibilities to influence the maternal metabolomics during pregnancy by specific diet or lifestyle and its possible influence on the future health of the offspring. Optimally, an intervention starting early in pregnancy would still have a window of opportunity to influence offspring growth and future health.

### Supplementary information

Supplementary tables

## Data Availability

Present informed consents do not allow archiving clinical or register data in open repositories. Data described in the manuscript, code book, and analytic code will be made available upon reasonable request and requests are subject to further review by the national register authority and by the ethical committees.
