# Supplementary material for: Early ascending growth is associated with maternal lipoprotein profile during mid and late pregnancy and in cord blood
Source: Int J Obes (Lond). 2023 Aug 17;47(11):1081–7. doi: 10.1038/s41366-023-01361-x (PMC10599999; doi:10.1038/s41366-023-01361-x)
Supplement: Supplementary file 1 — Supplementary tables [file 41366_2023_1361_MOESM1_ESM.docx]

**Supplementary tables:**

**Supplementary Table 1.** Means of the metabolites in the first trimester in growth profile groups (ascending n=41, intermediate n=138, descending n= 53)

| Metabolite | Ascending | Intermediate | Descending | Total |
| --- | --- | --- | --- | --- |
| PL ratio in XS-VLDL | 3.423 (0.046) | 3.436 (0.047) | 3.454 (0.047) | 3.437 (0.048) |
| TG ratio in M-LDL | 2.308 (0.201) | 2.220 (0.181) | 2.222 (0.154) | 2.236 (0.181) |
| TG in S-LDL | 0.034 (0.012) | 0.031 (0.010) | 0.032 (0.009) | 0.032 (0.010) |
| C ratio in S-VLDL | 3.566 (0.109) | 3.611 (0.104) | 3.616 (0.109) | 3.604 (0.107) |
| CE in S-VLDL | 3.029 (0.182) | 3.105 (0.163) | 3.113 (0.169) | 3.093 (0.170) |
| TG in XS-VLDL | 3.215 (0.140) | 3.152 (0.161) | 3.129 (0.152) | 3.158 (0.157) |
| TG ratio in IDL | 2.648 (0.158) | 2.580 (0.169) | 2.566 (0.150) | 2.589 (0.165) |
| TG ratio in L-LDL | 2.438 (0.190) | 2.362 (0.177) | 2.356 (0.149) | 2.374 (0.175) |
| C ratio in M-LDL | 4.153 (0.055) | 4.173 (0.045) | 4.180 (0.046) | 4.171 (0.048) |
| C ratio in S-LDL | 4.092 (0.067) | 4.114 (0.055) | 4.124 (0.058) | 4.112 (0.059) |
| TG ratio in S-LDL | 2.200 (0.202) | 2.122 (0.186) | 2.106 (0.166) | 2.132 (0.186) |
| C ratio in M-HDL | 3.872 (0.053) | 3.892 (0.044) | 3.895 (0.037) | 3.889 (0.045) |
| CE ratio in S-HDL | 3.423 (0.097) | 3.444 (0.089) | 3.467 (0.090) | 3.446 (0.092) |
| TG ratio in M-HDL | 1.797 (0.189) | 1.725 (0.199) | 1.691 (0.161) | 1.730 (0.191) |
| C ratio in S-HDL | 3.714 (0.072) | 3.737 (0.060) | 3.751 (0.062) | 3.736 (0.064) |
| MUFAFA | 3.375 (0.077) | 3.335 (0.144) | 3.301 (0.159) | 3.334 (0.141) |

**Supplementary table 2.** Means of the metabolites in the second trimester in growth profile groups (ascending n=46, intermediate n=160, descending n= 64)

| Metabolite | Ascending | Intermediate | Descending | Total |
| --- | --- | --- | --- | --- |
| C ratio in M-HDL | 3.795 (0.0769) | 3.831 (0.0584) | 3.817 (0.0526) | 3.822 (0.0619) |
| CE ratio in M-HDL | 3.553 (0.0929) | 3.597 (0.0696) | 3.577 (0.0658) | 3.585 (0.0747) |
| TG ratio in M-HDL | 1.998 (0.2303) | 1.871 (0.2082) | 1.900 (0.1631) | 1.899 (0.2069) |
| C ratio in XS-VLDL | 3.816 (0.0605) | 3.844 (0.0612) | 3.848 (0.0527) | 3.840 (0.0600) |
| CE ratio in S-VLDL | 3.085 (0.1511) | 3.132 (0.1621) | 3.150 (0.1552) | 3.129 (0.1595) |
| TG ratio in XL- HD | 1.742 (0.2164) | 1.642 (0.2068) | 1.642 (0.1544) | 1.659 (0.2002) |
| TG ratio in L-HDL | 2.006 (0.1837) | 1.920 (0.1540) | 1.937 (0.1302) | 1.938 (0.1568) |
| TG ratio in S-HDL | 2.014 (0.2086) | 1.920 (0.1895) | 1.964 (0.1661) | 1.946 (0.1903) |
| C in VLDL | 0.641 (0.1602) | 0.589 (0.1318) | 0.612 (0.1139) | 0.603 (0.1340) |
| PL in S-VLDL | 0.173 (0.046) | 0.153 (0.038) | 0.157 (0.033) | 0.157 (0.039) |
| TG in S-VLDL | 0.276 (0.085) | 0.235 (0.075) | 0.239 (0.059) | 0.243 (0.074) |
| FC ratio in XS-VLDL | 2.845 (0.0361) | 2.851 (0.0392) | 2.851 (0.0363) | 2.850 (0.0379) |
| L in L-VLDL | 0.304 (0.1447) | 0.245 (0.1365) | 0.251 (0.0967) | 0.257 (01310) |
| PL in L-VLDL | 0.067 (0.0353) | 0.054 (0.0321) | 0.055 (0.0221) | 0.056 (0.0309) |
| CE in L-VLDL | 0.044 (0.0231) | 0.034 (0.0198) | 0.035 (0.0135) | 0.036 (0.0194) |
| FC in M-VLDL | 0.091 (0.038) | 0.075 (0.033) | 0.076 (0.024) | 0.078 (0.033) |
| M-VLDL | 2.177 e^-8^ (0.94 e^-8^) | 1.778 e^-8^ (0.83 e^-8^) | 1.787 e^-8^ (0.58 e^-8^) | 1.848 e^-8^ (0.81 e^-8^) |
| PL in M-VLDL | 0.141 (0.054) | 0.118 (0.047) | 0.120 (0.034) | 0.122 (0.046) |
| C in M-VLDL | 0.196 (0.073) | 0.167 (0.061) | 0.172 (0.045) | 0.173 (0.060) |
| CE in M-VLDL | 0.116 (0.043) | 0.100 (0.034) | 0.104 (0.026) | 0.103 (0.035) |
| TG in M-VLDL | 0.304 (0.1191) | 0.251 (0.1131) | 0.253 (0.0817) | 0.261 (0.1090) |
| L in S-VLDL | 0.580 (0.142) | 0.520 (0.121) | 0.533 (0.102) | 0.533 (0.122) |
| TG in XS-VLDL | 0.158 (0.0432) | 0.140(0.0367) | 0.145(0.0349) | 0.144(0.0379) |
| TG ratio in S-LDL | 2.331 (0.1631) | 2.275(0.1551) | 2.278(0.1338) | 2.285(0.1526) |
| TG in IDL | 0.184 (0.0444) | 0.168(0.0396) | 0.177(0.0419) | 0.173(0.0413) |
| TG in L-LD | 0.170 (0.0397) | 0.157(0.0363) | 0.168(0.0397) | 0.162(0.0380) |
| TG in M-LDL | 0.089 (0.0210) | 0.082 (0.0192) | 0.088 (0.0212) | 0.085 (0.0202) |
| TG in S-LDL | 0.054 (0.0138) | 0.049 (0.0118) | 0.052 (0.0119) | 0.050 (0.0123) |
| FC ratio in IDL | 2.925 (0.0430) | 2.943 (0.0497) | 2.944 (0.0381) | 2.940 (0.0464) |
| XL-HDL | 7.134 e^-7^ (2.290 e^-7^) | 7.808 e^-7^ (2.290 e^-7^) | 8.333 e^-7^ (2.209 e^-7^) | 7.820 e^-7^ (2.225 e^-7^) |
| L in XL-HDL | 0.534 (0.1098) | 0.571 (0.1313) | 0.601 (0.1216) | 0.572 (0.1270) |
| PL in XL-HDL | 0.308 (0.0697) | 0.334 (0.0860) | 0.352 (0.0787) | 0.334 (0.0826) |
| C in XL- HDL | 0.274 (0.0667) | 0.297 (0.0794) | 0.317 (0.0758) | 0.298 (0.0775) |
| CE in XL-HDL | 0.207 (0.0504) | 0.225 (0.0605) | 0.241 (0.0586) | 0.226 (0.0592) |
| FC in XL-HDL | 0.083 (0.0240) | 0.091 (0.0287) | 0.097 (0.0272) | 0.091 (0.0279) |
| L-HDL | 1.970 e^-6^ (0.443 e^-6^) | 2.137 e^-6^ (0.511 e^-6^) | 2.210 e^-6^ (0.469 e^-6^) | 2.126 E^-6^ (0.495 e^-6^) |
| L in L-HDL | 0.795 (0.1283) | 0.841 (0.1406) | 0.862 (0.1274) | 0.838 (0.1367) |
| PL in- L-HDL | 0.447 (0.0760) | 0.477 (0.0850) | 0.488 (0.0762) | 0.475 (0.0823) |
| C in L-HDL | 0.455 (0.0970) | 0.492 (0.1067) | 0.508 (0.0961) | 0.490 (0.1037) |
| CE in L-HDL | 0.373 (0.0809) | 0.404 (0.0889) | 0.416 (0.0802) | 0.401 (0.0864) |
| FC-in L-HDL | 0.120 (0.0315) | 0.132 (0.0364) | 0.138 (0.0330) | 0.132 (0.0352) |
| TG-in M-HDL | 0.059 (0.0116) | 0.053 (0.0120) | 0.053 (0.0093) | 0.054 (0.0115) |
| C ratio in L-HDL | 3.872 (0.043) | 3.887 (0.040) | 3.892 (0.035) | 3.886 (0.040) |
| CE ratio in L-HDL | 3.632 (0.0374) | 3.646 (0.0333) | 3.648 (0.0295) | 3.644 (0.0335) |
| FC ratio in L-HDL | 2.418 (0.0601) | 2.439 (0.0651) | 2.449 (0.0531) | 2.438 (0.0621) |
| PL ratio in M-HDL | 3.928 (0.0335) | 3.916 (0.0329) | 3.926 (0.0306) | 3.921 (0.0328) |
| Serum TG | 1.053 (0.2177) | 0.960 (0.1970) | 0.985 (0.1537) | 0.982 (0.1937) |
| MUFA | 1.649 (0.2106) | 1.604 (0.1618) | 1.642 (0.1302) | 1.620 (0.1654) |
| TG in VLDL | 0.739 (0.2219) | 0.639 (0.2098) | 0.653 (0.1553) | 0.659 (0.2030) |
| HDL size | 2.418 (0.0153) | 2.424 (0.0186) | 2.428 (0.0180) | 2.424 (0.0182) |
| C in HDL | 1.029 (0.0959) | 1.071 (0.1064) | 1.083 (0.0946) | 1.067 (0.1031) |
| C in HDL2 | 0.798 (0.1117) | 0.849 (0.1206) | 0.860 (0.1053) | 0.843 (0.1171) |
| ApoBApoA1 | 0.455 (0.0830) | 0.424 (0.0660) | 0.433 (0.0563) | 0.431 (0.0678) |
| His | 0.0669 (0.0089) | 0.0704 (0.0080) | 0.0715 (0.0111) | 0.0701 (0.0091) |

**Supplementary table 3.** Means of the metabolites in the third trimester in growth profile groups (ascending n=43, intermediate n=170, descending n= 64)

| Metabolite | Ascending | Intermediate | Descending | Total |
| --- | --- | --- | --- | --- |
| C in HDL | 0.920 (0.1585) | 1.003 (0.1300) | 0.996 (0.1066) | 0.988 (0.1328) |
| C in HDL2 | 0.654 (0.1964) | 0.756 (0.1548) | 0.744 (0.1260) | 0.738 (0.1596) |
| L in L- HDL | 0.684 (0.1901) | 0.771 (0.1680) | 0.767 (0.1301) | 0.757 (0.1661) |
| PL in L-HDL | 0.378 (0.1131) | 0.433 (0.1002) | 0.427 (0.0790) | 0.423 (0.0994) |
| PL in XL-HDL | 0.246 (0.0985) | 0.295 (0.0957) | 0.295 (0.0750) | 0.287 (0.0931) |
| ApoA1 | 1.001 (0.0636) | 1.038 (0.0697) | 1.037 (0.0632) | 1.032 (0.0684) |
| XL-HDL | 5.832 e^-7^ (2.335 e^-7^) | 6.954 e^-7^ (2.467 e^-7^) | 6.982 e^-7^ (1.950 e^-7^) | 6.786 e^-7^ (2.364 e^-7^) |
| C in XL-HDL | 0.223 (0.0861) | 0.262 (0.0877) | 0.266 (0.0713) | 0.257 (0.0849) |
| CE in XL- HDL | 0.170 (0.0636) | 0.199 (0.0660) | 0.203 (0.0540) | 0.195 (0.0638) |
| FC in XL-HDL | 0.064 (0.0309) | 0.078 (0.0317) | 0.078 (0.0252) | 0.076 (0.0305) |
| C in L-HDL | 0.369 (0.1407) | 0.432 (0.1278) | 0.427 (0.0965) | 0.421 (0.1250) |
| CE in L-HDL | 0.302 (0.1153) | 0.354 (0.1056) | 0.350 (0.0795) | 0.345 (0.1030) |
| FC in L-HDL | 0.093 (0.0435) | 0.113 (0.0421) | 0.110 (0.0314) | 0.109 (0.0406) |
| L-HDL | 1.642 e^-6^ (0.587 e^-6^) | 1.919 e^-6^ (0.579 e^-6^) | 1.887 e^-6^ (0.436 e^-6^) | 1.870 e^-6^ (0.557 e^-6^) |
| FC in M-HDL | 0.073 (0.0234) | 0.083 (0.0187) | 0.080 (0.0178) | 0.081 (0.0196) |
| TG ratio in M-HDL | 2.305 (0.3771) | 2.166 (0.2623) | 2.213 (0.2188) | 2.199 (0.2778) |
| CE in XL-VLDL | 0.017 (0.0109) | 0.014 (0.0069) | 0.015 (0.0062) | 0.015 (0.0076) |
| L-VLDL | 1.209 e^-8^ (0.574 e^-8^) | 1.025 e^-8^ (0.379 e^-8^) | 1.034 e^-8^ (0.368 e^-8^) | 1.056 e^-8^ (0.415 e^-8^) |
| M-HDL | 2.021 e^-6^ (0.468 e^-6^) | 2.196 e^-6^ (0.413 e^-6^) | 2.135 e^-6^ (0.387 e^-6^) | 2.155 e^-6^ (0.420 e^-6^) |
| L in M-HDL | 0.603 (0.1139) | 0.647 (0.0934) | 0.633 (0.0890) | 0.637 (0.0968) |
| PL in M-HDL | 0.357 (0.0615) | 0.381 (0.0517) | 0.375 (0.0499) | 0.376 (0.0534) |
| C in M-HDL | 0.283 (0.0997) | 0.320 (0.0782) | 0.306 (0.0721) | 0.311 (0.0814) |
| CE in M-HDL | 0.225 (0.0862) | 0.256 (0.0686) | 0.243 (0.0626) | 0.248 (0.0709) |
| TG ratio in XL-HDL | 2.089 (0.3425) | 1.957 (0.3079) | 1.970 (0.2243) | 1.980 (0.2990) |
| TG ratio in L-HDL | 2.284 (0.3246) | 2.178 (0.2328) | 2.204 (0.1765) | 2.200 (0.2395) |
| HDL size | 2.403 (0.0232) | 2.414 (0.0212) | 2.414 (0.0167) | 2.412 (0.0209) |
| C in HDL3 | 0.457 (0.0250) | 0.466 (0.0260) | 0.470 (0.0263) | 0.466 (0.0262) |
| TG/PG ratio | 0.616 (0.1356) | 0.571 (0.1072) | 0.587 (0.0932) | 0.582 (0.1099) |
| Omega 6 FA | 3.366 (0.0832) | 3.405 (0.0873) | 3.390 (0.0734) | 3.395 (0.0845) |
| PUFAFA | 3.493 (0.0827) | 3.531 (0.0877) | 3.520 (0.0700) | 3.523 (0.0839) |
| LAFA | 3.191 (0.1074) | 3.236 (0.1132) | 3.218 (0.0950) | 3.225 (0.1092) |
| Ala | 0.330 (0.0334) | 0.339 (0.0309) | 0.346 (0.0352) | 0.339 (0.0325) |
| SM | 0.445 (0.0569) | 0.464 (0.0556) | 0.473 (0.0598) | 0.463 (0.0572) |

**Supplementary table 4.** Means of the metabolites in the third trimester in growth profile groups (ascending n=47, intermediate n=204, descending n= 94)

| Metabolite | Ascending | Intermediate | Descending | Total |
| --- | --- | --- | --- | --- |
| Lactate | 1.471 (0.3257) | 1.607 (0.3044) | 1.502 (0.2889) | 1.560 (0.3078) |
| VLDL size | 3.586 (0.0283) | 3.576 (0.0225) | 3.572 (0.0173) | 3.576 (0.0225) |
| Omega 3 FA | 0.206 (0.0542) | 0.220 (0.0617) | 0.213 (0.0550) | 0.216 (0.0590) |
| Citrate | 0.125 (0.0219) | 0.136 (0.0268) | 0.128 (0.0201) | 0.132 (0.0249) |
| XL-VLDL | 2.173 e^-10^ (2.14859 e^-10^) | 1.594 e^-10^ (0.968 e^-10^) | 1.475 e^-10^ (0.816 e^-10^) | 1.645 e^-10^ (1.193 e^-10^) |
| L in XL-VLDL | 0.021 (0.0201) | 0.015 (0.0093) | 0.014 (0.0079) | 0.016 (0.0113) |
| L in L-VLDL | 0.062 (0.0400) | 0.050 (0.0267) | 0.045 (0.0243) | 0.050 (0.0286) |
| L-VLDL | 1.09456 e^-9^ (0.751 e^-9^) | 0.869 e^-9^ (0.489 e^-9^) | 0.782 e^-9^ (0.436 e^-9^) | 0.87597 e^-9^ (0.526 e^-9^) |
| TG in VLDL | 0.227 (0.0901) | 0.200 (0.0665) | 0.192 (0.0630) | 0.201 (0.0698) |
| TG/PG ratio | 0.357 (0.1371) | 0.323 (0.1307) | 0.292 (0.1107) | 0.319 (0.1277) |
| PUFA | 0.836 (0.1042) | 0.839 (0.1036) | 0.831 (0.0931) | 0.836 (0.1006) |
| DHA | 0.088 (0.0269) | 0.094 (0.0270) | 0.092 (0.0253) | 0.093 (0.0265) |
| Gln | 0.286 (0.0445) | 0.283 (0.0469) | 0.265 (0.0428) | 0.278 (0.0460) |
| His | 0.076 (0.0124) | 0.080 (0.0124) | 0.082 (0.0128) | 0.080 (0.0126) |
